# Cognitive complaints in schizophrenia:relationship with clinical symptoms, stigma and insight

**DOI:** 10.1192/j.eurpsy.2024.1577

**Published:** 2024-08-27

**Authors:** S. Ajmi, M. Bouhamed, R. Ouali, S. Hentati, I. Feki, R. Sallemi, J. Masmoudi

**Affiliations:** Psychiatry A, Hedi Chaker University Hospital, Sfax, Tunisia

## Abstract

**Introduction:**

In addition to the classic clinical symptoms, patients with schizophrenia suffer from cognitive difficulties, self-stigma and poor insight.

**Objectives:**

This study aims to evaluate the impact of stigma, symptom severity, and insight on subjective cognitive complaints in patients with schizophrenia.

**Methods:**

This is a cross-sectional, descriptive and analytical study carried out on 72 stabilized patients followed at the post-cure psychiatry consultation ‘A’ at the CHU Hédi Chaker in Sfax diagnosed with schizophrenia according to the DSM 5 criteria.

We used the schedule for the Assessment of Insight–Expanded Version(SAI-E) to assess clinical insight, The Internalized Stigma of Mental Illness (ISMI) scale for the assessment of internalized stigma, the Subjective Scale to Investigate Cognition in Schizophrenia (SSTICS) scale to determine subjective cognitive complaints and the Positive and Negtive Syndroms Scale (PANSS) to assess positive and negatives symptoms.

**Results:**

The average age of the patients was 46.83 ± 11.6 years, with a sex ratio (M/F) of 2. In our study, 48.5% of the patients were single, 52.8years,% were smokers and 23 6% consumed alcohol. The level of education did not exceed the primary level for 44.4% of the patients. The average age of disease onset was 24.56 ± 5, 82. Our participants had an average score of 25 on the SSTICS total score and 20.1 on the SAI-E. The median ISMI total score and PANSS total score were 2.45 and 46respectively

The predominant negative symptoms(p=0.003), stigma(p=0.009), and insight (p<10-3)were significant factors associated with increased cognitive complaints.

**Conclusions:**

In schizophrenia, the combination of cognitive difficulties, self-stigma with a low insight makes the management of these patients more difficult.

**Disclosure of Interest:**

None Declared

